# Gene age and genome organization in *Escherichia coli* and *Bacillus subtilis*

**DOI:** 10.3389/fmicb.2025.1512923

**Published:** 2025-06-18

**Authors:** Carsten Jers, Hrvoje Mišetić, Vaishnavi Ravikumar, Abhroop Garg, Damjan Franjević, Tomislav Domazet-Lošo, Ivan Mijakovic

**Affiliations:** ^1^Novo Nordisk Foundation Center for Biosustainability, Technical University of Denmark, Kongens Lyngby, Denmark; ^2^Laboratory Evolutionary Genetics, Division of Molecular Biology, Ruder Bošković Institute, Zagreb, Croatia; ^3^Division of Biology, University of Zagreb, Zagreb, Croatia; ^4^School of Medicine, Catholic University of Croatia, Zagreb, Croatia; ^5^Systems and Synthetic Biology Division, Department of Life Sciences, Chalmers University of Technology, Gothenburg, Sweden

**Keywords:** genomic phylostratigraphy, horizontal gene transfer, operon structure, prophages, transcription regulators

## Abstract

Using genomic phylostratigraphy, we examined the organization of *Escherichia coli* and *Bacillus subtilis* genomes from the perspective of evolutionary age of their genes. Phylostratigraphy analysis classifies individual genes into age-related bins, called phylostrata. Based on this analysis, several common features emerged in the genomes of the two model bacteria. More recent genes tend to be shorter and are expressed less frequently, or only in specific conditions. In terms of genomic location, new genes are enriched in areas containing prophages, suggesting a link with horizontal gene transfer. Interestingly, while most bacterial transcription regulators belong to the oldest phylostrata, they regulate expression of both older and more recent genes alike. A large fraction of bacterial operons contains genes from different phylostrata. This suggests that newer genes are integrated in the existing framework for regulating gene expression, and that the establishment of new regulatory circuits typically do not accompany acquisition of new genes. One striking difference between *E. coli* and *B. subtilis* genomes was observed. About 87.0% of all *E. coli* genes belong to the evolutionary oldest physlostratum. In *B. subtilis*, this number is only 71.8%, indicating a more eventful evolutionary past in terms of acquisition of new genes, either by gene emergence or by horizontal transfer.

## 1 Introduction

Understanding the origins and evolution of genes remains one of the central challenges in evolutionary biology. A fundamental question is how new genetic material arises, becomes integrated into existing cellular networks, and contributes to cell fitness and diversity. While ancient genes underpin essential cellular functions, new genes can introduce novel traits and adaptive advantages. Exploring the mechanisms and dynamics of gene emergence and loss is therefore key to understanding the evolution of genomes over time. The oldest genes present in the extant genomes are the ones there were also present in the last universal common ancestor (LUCA; Mushegian, 2008). Newer genes are the ones that emerged later. Besides a variety of duplication-based mechanisms (Tautz and Domazet-Lošo, 2011), new genes could emerge from non-coding DNA sequences through random mutations (Neme and Tautz, 2014). While it has been proposed that this type of gene emergence is common in all species, the frequency of this phenomenon probably varies a lot, and is difficult to assess quantitatively (Light et al., 2014). Recently, Iyengar and Bornberg-Bauer (2023) developed a mathematical model that suggests that genes are lost much more rapidly than they emerge, and that new genes preferentially arise in transcribed regions. This concept is well-illustrated by a recent study of human proto genes (Grandchamp et al., 2022), suggesting that new genes tend to “capture” regulatory sequences in their vicinity, such as introns, promoter motifs, enhancers. Proto genes that survive the initial selection tend to grow by gaining new functional domains. While comparative genomics has provided strong evidence for the phenomenon of *de novo* gene emergence in various organisms, experimental validation of gene emergence is obviously challenging. To experimentally illustrate the selection process following “gene birth,” Knopp et al. (2019) constructed plasmid libraries with short randomly generated open reading frames (ORFs) and expressed them in *Escherichia coli*. They demonstrated that short peptides offering a fitness benefit during exposure to aminoglycoside antibiotics could be effectively selected by bacterial cells.

Genomic phylostratigraphy is a computational method for studying genome evolution based on estimating evolutionary age of individual genes (Domazet-Lošo et al., 2007). For any species to be analyzed, the first step is to create a consensus phylogeny tree. In this species-centered tree, each node is named a phylostratum (PS). Each PS is then populated with individual genes whose founders emerged at that specific node. From the whole genome perspective, this means that all genes get distributed in phylostrata corresponding to their evolutionary age. In the early evolutionary history, the first few PS correspond to deep, shared ancestry (e.g., LUCA, Bacteria) and are therefore common across different bacterial species such as *E. coli* and *B. subtilis*. Subsequent PS become increasingly species-specific as bacterial lineages diverge. Phylostratigraphy has proven to be a powerful method for explaining macro-evolutionary phenomena. Certain complex functions that emerged at some point in time tend to involve large sets of genes, which then cluster to a specific PS. Hence, phylostratigraphy was used to demonstrate that cancer-related genes could be coupled to the emergence of multicellularity (Domazet-Lošo and Tautz, 2010a), and that the expression of genes during ontogenic development of metazoa follows an age-specific pattern (Domazet-Lošo and Tautz, 2010a). The latter study has been particularly noted for providing direct evidence for the hourglass theory of development (Casci, 2011). There has been some debate about the reliability of sequence similarity search algorithms underlying phylostratigraphic approach (Moyers and Zhang, 2015, 2016; Domazet-Lošo et al., 2017, 2024). However, the statistical mapping of well-studied functional data on phylogenies repeatedly demonstrates that classical sequence similarity searches accurately recover macroevolutionary information (Domazet-Lošo et al., 2017, 2024; Xia et al., 2025).

It is known that bacterial genomes undergo extensive horizontal gene transfer (Arnold et al., 2022). This evolutionary process is particularly prominent in mixed bacterial communities (Brito, 2021). Mobile genetic elements, such as plasmids and phages, have been recognized as the main driver of horizontal gene transfer (Lang et al., 2017). Since phylostratigraphy was initially developed for metazoa, i.e., species with predominantly vertical mode of evolution, it was not initially clear how well the method would perform in analyzing bacterial genome evolution. However, it turned out to work remarkably well. Using genomic phylostratigraphy, Futo et al. (2021) demonstrated that the development of *Bacillus subtilis* biofilms recapitulates phylogeny at the expression level. This finding suggested that the ontogeny of bacterial biofilms is a developmental process similar to that of e.g., metazoans or plants (Koska et al., 2024). Similarly, phylostratigraphy has been used to characterize the development of different morphotypes of *Borreliella* (syn. Borrelia) *burgdorferi* (Corak et al., 2023). Based on the assumption that bacterial sporulation is also a true developmental phenomenon, phylostratigraphy has been successfully used to predict new sporulation genes, among uncharacterized genes in sporulation-related phylostrata (Shi et al., 2020). This validated the earlier proposition that phylostratigraphy could be a useful tool in genome mining pipelines, as previously suggested (Mijakovic, 2020).

Bacterial genomes are large circular DNA molecules, in which the origin of replication (and terminus of replication, at 180° with respect to origin) plays a major organizational role (Duigou and Boccard, 2017). Each chromosome arm between the origin of replication and replication terminus consists of two regions, a non-structured region and a so-called macrodomain (Valens et al., 2004). The “Right” and “Left” macrodomains are defined by their incapacity to interact with each other, a phenomenon linked to high level structural organization of the bacterial genome. The position of genes with respect to the bacterial origin of replication has a deep significance. Genes near the replication origin tend to be more highly expressed (Ying et al., 2014; Kosmidis et al., 2020; Lato and Golding, 2020). Gene essentiality is also high near the origin of replication (Kosmidis et al., 2020; Lato and Golding, 2020). Genes farther away from the origin are more prone to molecular changes, such as substitutions, recombination events and genomic rearrangements (Lato and Golding, 2021). In *B. subtilis*, during asymmetric division leading up to spore formation, chromosomal location governs the timing of expression of sporulation genes (Zupancic et al., 2001).

In this report, we used genomic phylostratigraphy to examine the relationship between gene age and chromosomal organization in two model bacterial species, *B. subtilis* and *E. coli*. We hypothesized that differences in lifestyle and ecology between *B. subtilis* (predominantly soil-dwelling, sporulating bacterium) and *E. coli* (a facultative anaerobe associated with animal hosts) may have influenced their evolutionary paths and their propensity to acquire new genes. We further hypothesized that bacterial genome dynamics, including rate of gene acquisition, genomic placement of new genes, their transcriptional regulation, and their lateral mobility, might exhibit distinct patterns compared to those observed in eukaryotes.

## 2 Materials and methods

### 2.1 Phylostratigraphic analysis

Phylostratigraphic maps were generated based on protein sequence data downloaded from the Uniprot homepage (UniProt Consortium, 2023), as described before (Ravikumar et al., 2018; Shi et al., 2020; Futo et al., 2021). The genomes to produce the phylostratigraphic tree and the protein sequences analyzed for *B. subtilis* 168 and *E. coli* K12 are detailed in Supplementary Data Sheets 1, 2, respectively. The consensus phylogenetic tree covering the divergence from the last common ancestor of cellular organisms to the *B. subtilis* was done as described previously (Domazet-Lošo et al., 2007; Domazet-Lošo and Tautz, 2010b). For *B. subtilis*, 4,177 of 4,197 proteins were assigned a phylostratigraphic age and in case of *E. coli* 4,279 of 4,306 proteins were assigned an age. The taxon ID from NCBI was used except for groups A, B, C (1708685, 1708686, 1708687, 1708688, 1708689).

### 2.2 Data acquisition

To correlate various protein properties with phylostratigraphic age, global datasets were extracted. For both *B. subtilis* and *E. coli*, gene coordinates and the corresponding protein lengths were obtained from the BioCyc database (Karp et al., 2019). For *B. subtilis*, lists of genes located within annotated prophage regions (hereafter referred to as prophage genes), as well as data on operon structure, transcriptional regulation, and proteins involved in sporulation, competence, and biofilm formation were downloaded from the SubtiWiki v.3 database (Zhu and Stülke, 2018). Lists of genes expressed at low and high levels were obtained from a transcriptome study by Nicolas et al. (2012). For *E. coli*, a list of prophage genes was derived from a study of Wang et al. (2010). Lists of operon structure and transcriptional regulation was obtained from RegulonDB (Santos-Zavaleta et al., 2019). Finally, *E. coli* proteins implicated in biofilm formation was derived from BioCyc based on associated Gene ontology (GO) terms (Karp et al., 2019). The compiled data for *B. subtilis* and *E. coli* is available in Supplementary Data Sheets 3, 4, respectively. Calculation of the Spearman' correlation coefficient was done using the function CORREL in Excel (Microsoft) as was the calculation of *p*-value for Chi-square test using the CHISQ.TEST function.

### 2.3 Circular visualization of genomes

CiVi (Circular visualization for microbial genomes; Overmars et al., 2015) was used for the purpose of generating circular maps to represent positions of gene clusters on the genomes of *B. subtilis* 168 and *E. coli* K12. Groups of genes that belong to the same PS were imported and the data was displayed as position of the “genes on the plus strand” in the form of a single concentric circle. This was repeated subsequently for each PS individually. For each species, for the innermost ring, “coordinates” was chosen under the data display option, with the origin of replication denoted as zero.

### 2.4 Functional annotation

Functional annotation analysis of the proteins falling under different phylostrata in *B. subtilis* 168 and *E. coli* K12 was performed using DAVID (Huang da et al., 2009a,b). Phylostrata 1–5 of *B. subtilis* 168 and *E. coli* K12 fall under same groups (phylogeny). Hence, two groups were made (phylostrata 1–5 and 6–15) in case of *B. subtilis* and the analysis was performed overall on these two groups, using the DAVID Bioinformatics Resources. UniProt accession IDs were submitted as a gene list for this purpose. Functional annotation charts/tables using the categories GOTERM_BP_DIRECT, GOTERM_MF_DIRECT, and KEGG_PATHWAY were generated. Default parameters such as count threshold of 2 and *P*-value score of 0.1 were used. The same was done for *E. coli* wherein phylostrata 1–5 and 6–11 were grouped. For reference, the results of the analysis done for proteins in the individual phylostrata is presented (Supplementary Data Sheet 5).

## 3 Results

The bacterial species studied were selected based on their differing habitats, with *B. subtilis* being a predominantly soil-dwelling, sporulating bacterium, and *E. coli* a facultative anaerobe associated with animal hosts. Additionally, they were chosen because they are among the best-characterized bacterial models, with early selection driven by their biological properties (such as sporulation and rapid growth, respectively), followed by extensive development of genetic tools and comprehensive genomic resources. For *E. coli*, we specifically focused on the non-pathogenic laboratory K-12 strain, which serves as a standard reference genome and minimizes confounding variation associated with pathogenicity.

### 3.1 Phylostratigraphy maps of *E. coli* and *B. subtilis*: general features

**Figure 1:**
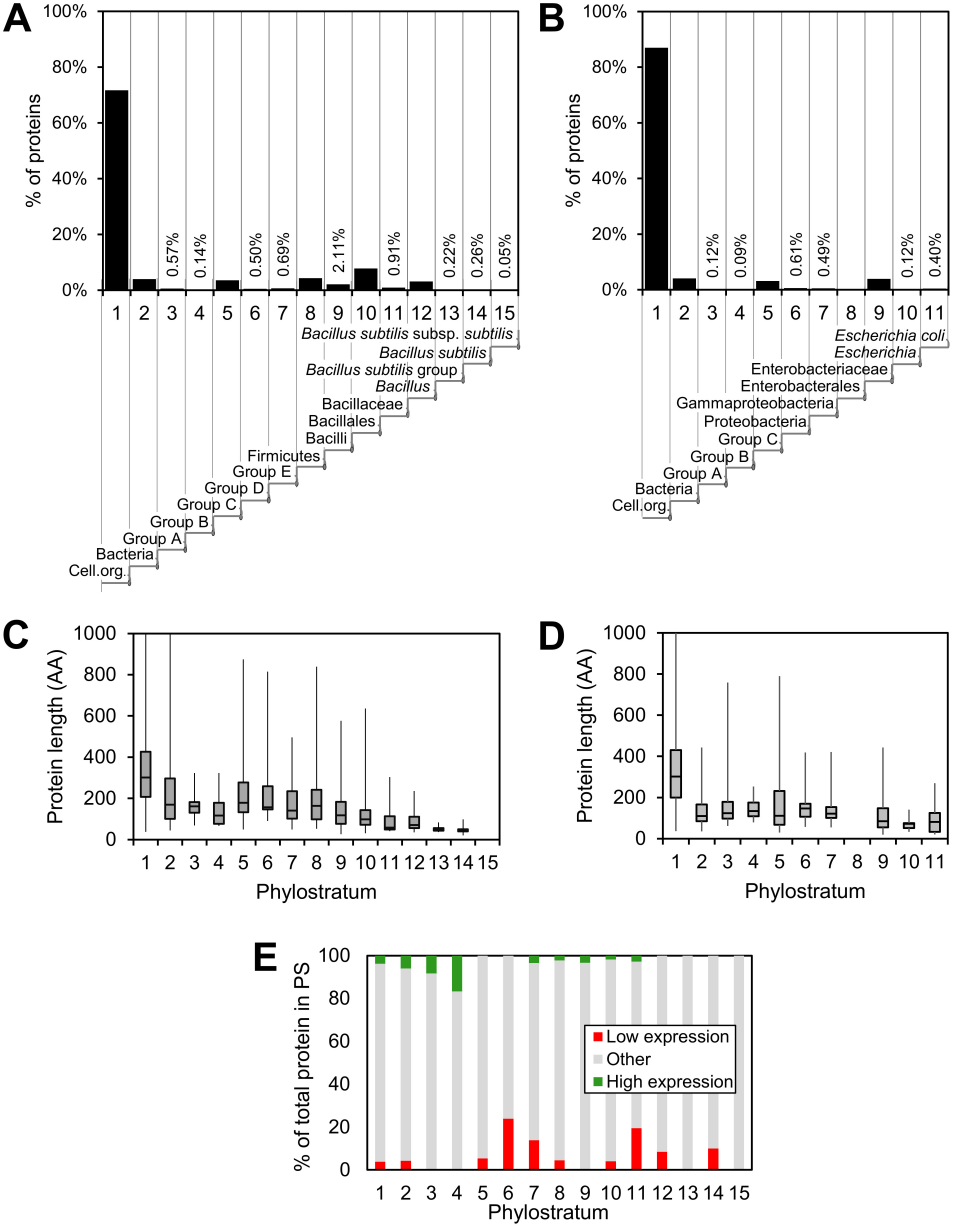
Distribution, protein length and expression within the various phylostrata. Distribution of proteins in respective phylostrata for **(A)** *B. subtilis* and **(B)** *E. coli*. The fraction of all proteins in the specific phylostrata is given (%) and an indication of the evolutionary trajectory is given below the phylostratum number. The average protein length in respective phylostrata is shown in a box plot for *B. subtilis* **(C)** and *E. coli* **(D)**. In **(E)**, the distribution of *B. subtilis* proteins encoded by genes that exhibit high (green) and low (red) expression within each phylostrata is shown.

Phylostratigraphy maps of *B. subtilis* and *E. coli* were constructed as previously described (Ravikumar et al., 2018; Shi et al., 2020; Futo et al., 2021; Figures 1A, B). In both bacteria, a large majority of genes clusters in the oldest PS. This phenomenon was more pronounced in *E. coli*, where 87.0% of genes belonged to PS1, whereas in *B. subtilis* that fraction was considerably lower, only 71.8%. This was a higher fraction than reported in the domain of Eukarya, where for example in humans about 38% of proteins belong to PS1 (Domazet-Lošo and Tautz, 2010a). Gene age was found to be inversely correlated to gene length in both bacteria (Figures 1C, D), in accord with the theory of “gene birth” from short ORFs (Neme and Tautz, 2014). A previous proteomics study indicated that “younger” bacterial genes are less expressed (Ravikumar et al., 2018). It was possible for us to re-examine this proposition by using an exhaustive transcriptome dataset from Nicolas et al. (2012). This study investigated transcriptional responses of *B. subtilis* in >100 different growth conditions. Genes that consistently showed either the lowest or the highest expression levels in all conditions were identified. Using this criterion, total genes in each PS were then categorized as possessing a “high” expression level or “low expression level,” while those not belonging to these two categories were labeled as “other” (Figure 1E). Based on these expression indicators from the transcriptome data (Nicolas et al., 2012), it was evident that for all PS, the majority of genes belonged to the “other” category, indicating intermediate or variable transcription levels. However, a trend was observed in which PS 1–4 contained a higher fraction of highly expressed genes, whereas PS 5–14 had a greater proportion of genes in the low expression category. It should be noted that PS3 and PS4 that exhibited the highest fraction of highly expressed genes consist of only 24 and 6 proteins, respectively. The very small PS15 (comprising 0.05% of the genome) did not contain any genes in either the “high” or “low” categories.

### 3.2 Location of new genes in bacterial genomes is not governed by distance to origin of replication

**Figure 2:**
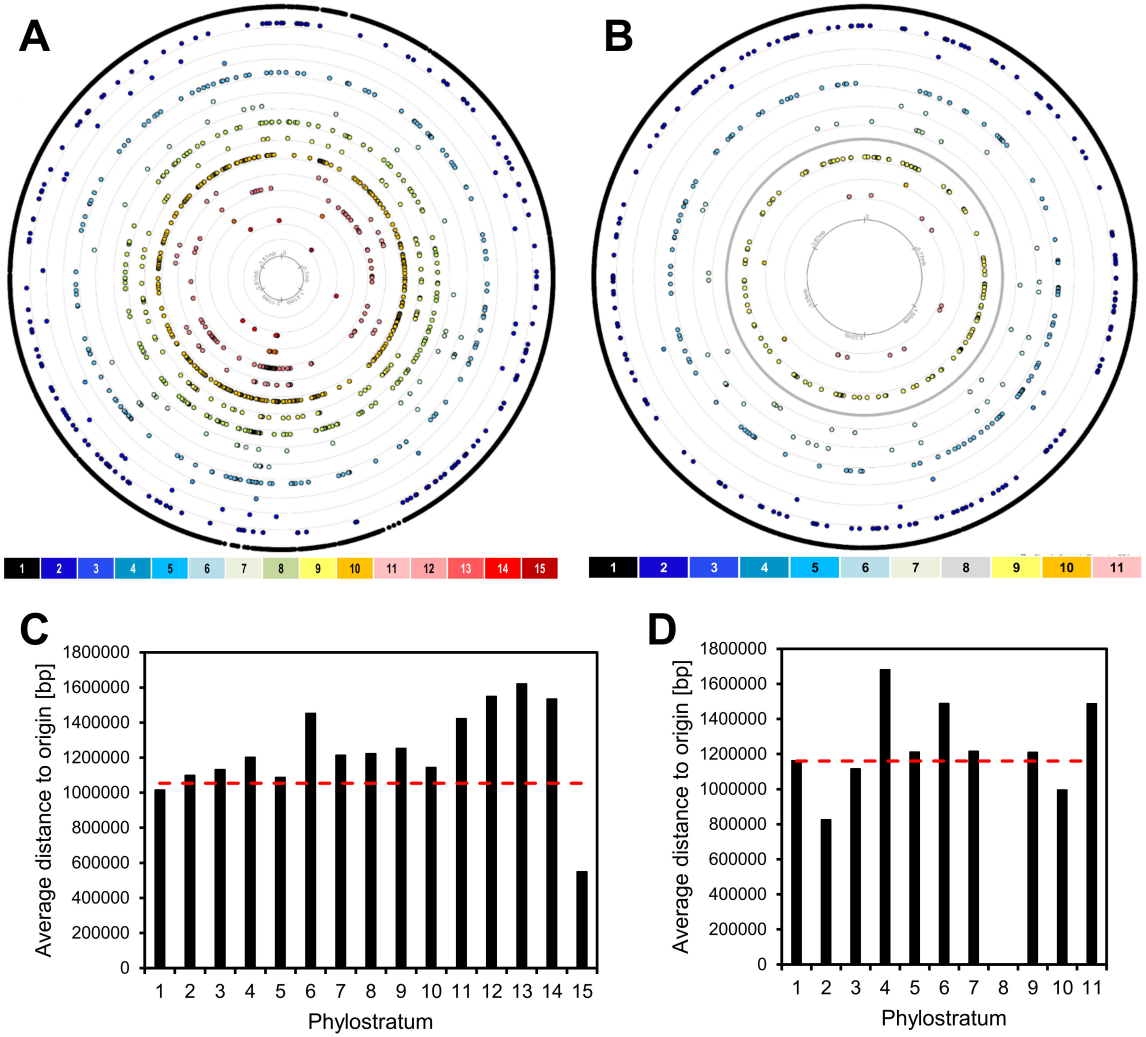
Genomic localization of genes. The genes of *B. subtilis* **(A)** and *E. coli* **(B)** are divided into 15 and 11 phylostrata (PS), depicted by concentric rings. Each dot in a phylostratum ring represents one protein-encoding gene. The rings are numbered 1–15, to be read from outside to inside. The innermost ring indicates the coordinates on the genome. The origin of replication is positioned at the top center of each circular genome map. The average distance to the origin of replication of genes within each of the phylostrata is shown for *B. subtilis* **(C)** and *E. coli* **(D)**. The dotted red line represents the length corresponding to even distribution of genes.

**Figure 3:**
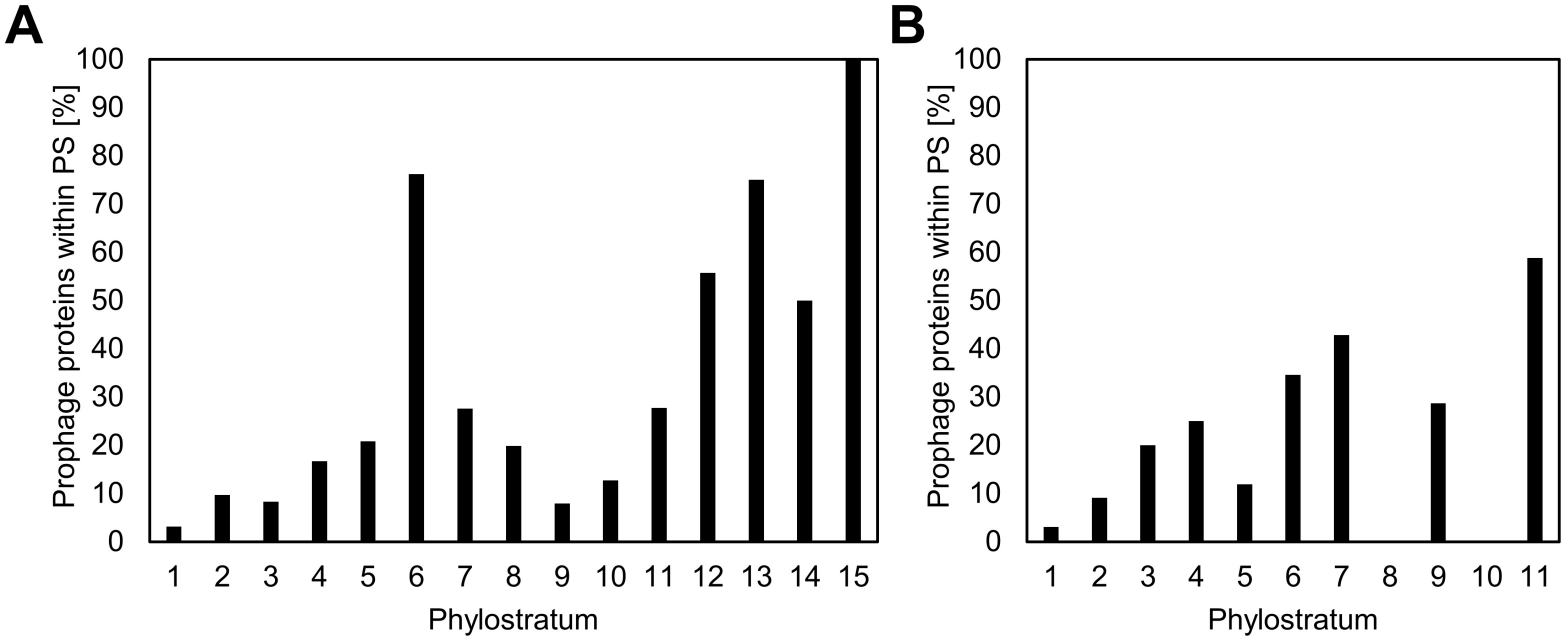
Enrichment of genes belonging to newer phylostrata in prophages. The fraction of prophage genes in the different phylostrata for *B. subtilis* **(A)** and *E. coli* **(B)** is presented. While there is a general trend of increased enrichment in newer genes, some phylostrata (e.g., PS6) contain relatively few genes, which may exaggerate apparent enrichment patterns and should be interpreted with caution.

High expression levels and gene essentiality are known to gravitate to the origin of replication in the bacterial chromosomes (Ying et al., 2014; Kosmidis et al., 2020; Lato and Golding, 2020). Since the newer genes are less strongly expressed and are most often involved in non-essential specialized functions (Ravikumar et al., 2018), we asked whether they would cluster toward the opposite end of the chromosome, the replication terminus. Although the precise locations of the origin and terminus of replication are typically inferred from sequence composition features such as GC skew (Grigoriev, 1998), these estimates are generally robust and sufficient for broad-scale analyses of gene distribution. In Figures 2A, B, the location of each gene on the respective genome maps of *B. subtilis* and *E. coli* is shown, color coded with respect to PS. Average distances from replication origin for genes in each PS show a weak inverse correlation (Spearman's correlation coefficient of 0.22) with gene age (Figures 2C, D). When prophage genes are excluded from the analysis, this weak correlation disappears completely (Spearman's correlation coefficient of −0.19; Supplementary Figure 1). There is also no evident enrichment of younger genes in “Right” vs. “Left” chromosome arm, nor in non-structured regions vs. macrodomains. However, we identified prophages as one genomic feature that shows significant enrichment in more recent genes, both in *E. coli* and *B. subtilis* (Figure 3). An alternative illustration of the genome organization, showing genes as boxes color-coded with respect to PS (Supplementary Figures 2A, B), illustrates the prophage regions (marked in red). The number of prophage genes constitute 8.3% and 5.2% in *B. subtilis* and *E. coli*, respectively. When quantifying the fraction of prophage genes in the different phylostrata (Figures 3A, B), it should be noted that certain phylostrata, such as PS6, contain relatively few genes, which could exaggerate apparent enrichment. Nevertheless, it was, evident that PS1 genes are underrepresented in prophages, and prophages are generally enriched in newer genes.

### 3.3 New genes tend to integrate into existing operons

**Figure 4:**
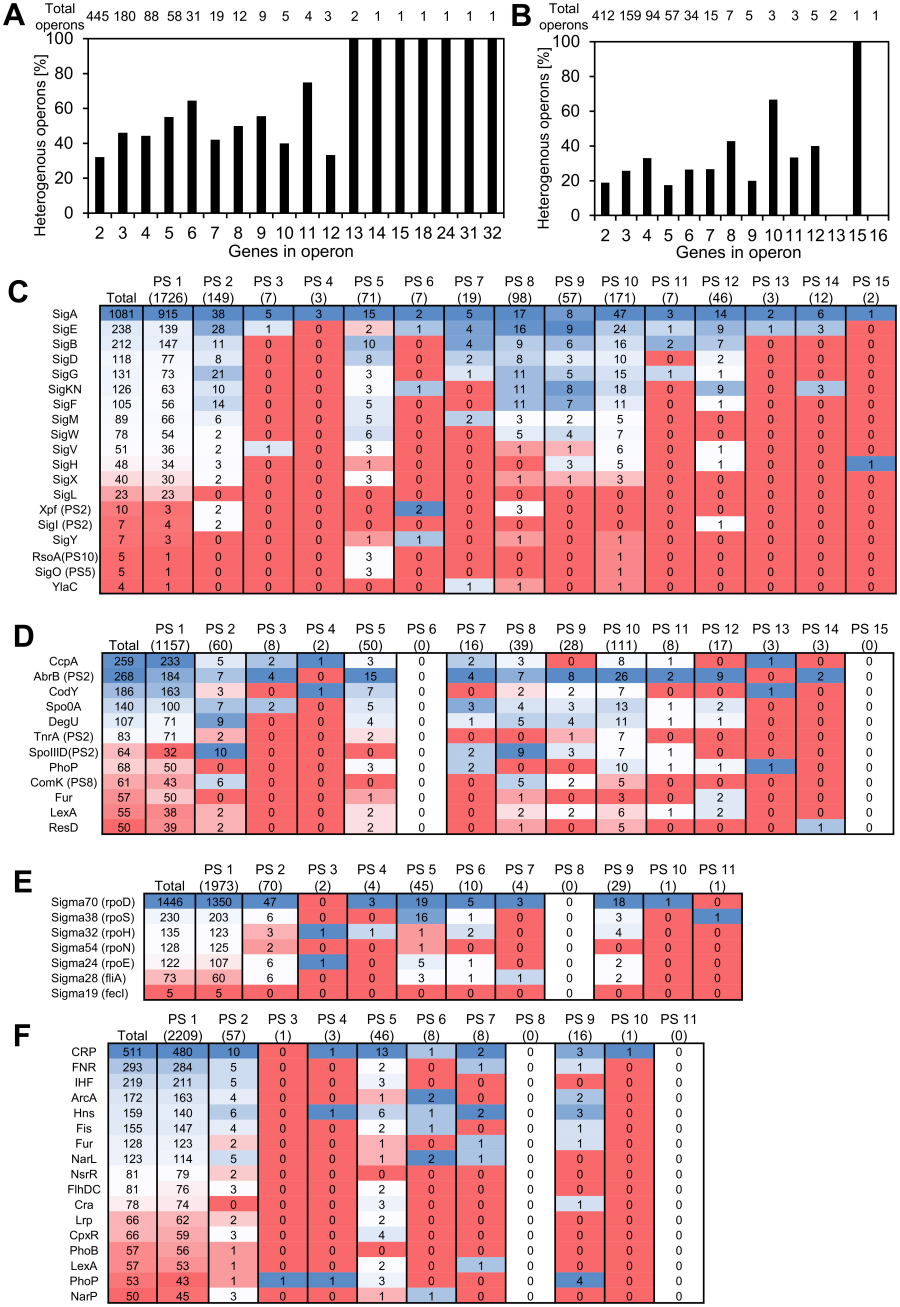
Transcriptional regulation and PS composition of operons. **(A, B)** Heterogenous operons as defined by presence of genes from different phylostrata in the same operon for *B. subtilis* **(A)** and *E. coli* **(B)**. **(C–F)** Heatmaps showing the number of genes in each phylostratum regulated by sigma factors **(C, E)** and global transcription regulators **(D, F)** in *B. subtilis* **(C, D)** and *E. coli* **(E, F)**. For each regulatory group, the distribution of regulated proteins in respective phylostrata is shown. Cells are color-coded using a blue–white–red gradient to emphasize relative gene counts across categories. These heatmaps are intended as a visual summary of count data and do not represent statistical significance or enrichment. For *B. subtilis*, regulators that do not belong to PS1 are indicated; in *E. coli*, all global regulators fall within PS1. For *B. subtilis*, it is noted when transcriptional regulators did not belong to PS1 (all global regulators in *E. coli* belonged to PS1).

Most bacterial genes are organized in operons, which are the basic transcription control units, with several genes expressed from one common promoter. With the data on evolutionary age of genes at hand, and given the operonic structure of bacterial genomes, we asked whether new genes typically form new transcriptional units, or if they get incorporated in preexisting operons. To address this question, we defined heterogenous operons as operons consisting of genes from two or more different phylostrata. For *B. subtilis*, 862 operon structures were reported in SubtiWiki and for *E. coli*, 798 operons were found in RegulonDB. Figures 4A, B shows the fraction of heterogenous operons for *E. coli* and *B. subtilis*, distributed per operon length, expressed as number of genes in operon. A significant fraction of all operons contains genes from two or more phylostrata, and this fraction increases with operon length. This is particularly evident in *B. subtilis*, where all operons with 13 or more genes were found to be heterogenous. While we did not systematically analyze genomic islands in this study, we did assess gene enrichment within annotated prophage regions. Although prophages are indeed enriched in newer genes, they account for a relatively small portion of the genome. Thus, the majority of new genes are located outside of prophages and often appear within annotated operons, suggesting that integration into existing transcriptional units is a common fate for new genes in these species. We then tried to probe what functionalities were introduced in the pre-existing operons. When we considered the operons consisting of genes with known functions, it seemed evident that the most common event was introduction of proteins that regulated one or more proteins in the operons by modulation either transcription or translation or by protein-protein interaction (Supplementary Table 1). There were also some examples of newer genes that were more difficult to rationalize. These included subunits of heterooligomeric proteins, an anti-toxin and even a protein reported to be essential. In general, most of the homogeneous operons contain genes from PS1. There exist only 11 non-PS1 homogenous operons in *B. subtilis* and 13 such operons were found in *E. coli*. Interestingly, several of these more recent homogenous operons appear to play a role in developmental phenomena such as sporulation in *B. subtilis* (Table 1) and biofilm formation in *E. coli* (Table 2).

**Table 1:** Homogenous, non-PS1 operons in *B. subtilis*.

**Operon**	**Phylostratum**	**Function**
*fbpA, fbpB*	12	Iron starvation
*yorB, yorC*	12	Unknown, SP-β prophage
*spoIISA, spoIISB*	10	Toxin-antitoxin, sporulation
*ykzB, ykoL*	10	Unknown
*yppD, yppE*	10	Unknown
*yxcE, yxcD*	10	Unknown
*spoVID, ysxE*	8	Sporulation
*cotB, ywrJ*	8	Sporulation
*sigI, rsgI*	2	Sigma factor/anti-sigma factor, heat shock
*gpr, spoIIP*	2	Sporulation/Germination
*yuzM, yusN*	2	Sporulation

**Table 2:** Homogenous, non-PS1 operons in *E. coli*.

**Operon**	**Phylostratum**	**Function**
*yffM, yffN*	11	Unknown, Prophage cpz55
*yffQ, yffR*	11	Unknown, Prophage cpz55
*yadM, yadL, yadK, yadC*	9	Biofilm, cryptic under normal laboratory conditions
*ydfA, ydfB, ydfC*	9	Unknown, Qin prophage
*kilR, ydaE*	9	Inhibitor of FtsZ, killing protein, Rac prophage
*ydaG, ydaF*	9	Unknown, Rac prophage
*yhaB, yhaC*	9	Unknown
*yjbL, yjbM*	9	Unknown
*ymcE, gnsA*	9	Cold shock protein; predicted regulator of phosphatidylethanolamine synthesis
*yfdP, yfdQ*	6	Unknown, Prophage CPS-53
*ynfO, ydfO*	5	Unknown, Qin prophage
*mokC, hokC*	2	Gef toxin, interferes with membrane function
*mqsR, mqsA*	2	Toxin-antitoxin, biofilm, persistence

### 3.4 There is no age correlation between genes and regulators that govern their expression

The operonic organization of bacterial genomes depends on transcriptional regulators that govern expression from different promoters. Out of the 194 proteins annotated as transcriptional regulators in *B. subtilis* on SubtiWiki, a majority (163; 84%) belonged to PS1 (Supplementary Figure 3A). Similarly, in *E. coli* we observed that 193 of 207 (93%) transcriptional regulators reported in RegulonDB belonged to PS1 (Supplementary Figure 3C). Since it was clear from the operon structure that most of the new genes land in existing operons, we investigated how this reflects on the relationship between the evolutionary age of genes and the age of transcription regulators that govern their expression. Among the evolutionary younger transcriptional regulators in *B. subtilis* were for example the competence regulator ComK (PS8; van Sinderen et al., 1994), its repressor Rok (PS10; Hoa et al., 2002), GerR involved in sporulation (PS8; Kuwana et al., 2005) and the master activator of flagellar biosynthesis SwrAA/1 (PS10; Calvio et al., 2005). We asked whether these evolutionary younger transcriptional regulators would preferentially regulate other younger genes. To assess this, we divided regulated proteins into three groups based on whether they are regulated by a transcriptional regulator belonging to either phylostratum 1, 2, or above 2 (Supplementary Figure 3). There was a weak tendency for the transcriptional regulators belonging to PS1 to preferentially regulate evolutionary older proteins (Supplementary Figures 3C, D). While these differences were statistically significant (Chi-square test *p*-value of 0.001 and 0.009 for *B. subtilis* and *E. coli*, respectively) it was observed that evolutionary younger transcriptional regulators can also adopt transcriptional control of older genes. For major players in regulating gene expression, the sigma factors and global transcriptional regulators, we created an overview of age correlation with the genes under their regulation by making heat maps (Figures 4C–F). In *B. subtilis* (Figures 4C, D), it became apparent that several sigma factors including SigE and SigK, known to be involved in regulation of sporulation (Haldenwang et al., 1981; Stragier et al., 1989), regulate a relatively higher number of proteins from newer phylostrata (PS2, 7, 8, 9 and PS8, 9, 12, respectively; Figure 4C). Among the regulated genes with a known function in these phylostrata, most were involved in various aspects of the sporulation process. Among the global transcriptional regulators, especially AbrB and to a lesser extent SpoIIID stood out (Figure 4D). For AbrB, regulated genes in the recent phylostrata were involved in sporulation, antibacterials biosynthesis and a substantial number of genes were of unknown function. For SpoIIID, mainly genes involved in sporulation were observed, consistent with the known regulatory role of SpoIIID (Kunkel et al., 1989). For *E. coli* it seemed less pronounced, that specific transcriptional regulators would preferentially regulate newer genes. That said, the sigma factor 38 (RpoS) showed an enrichment in PS5 genes (Figure 4E). Sigma factor 38 is induced upon entry into stationary phase and functions as a master regulator of the general stress response (Weber et al., 2005). Among PS5 genes regulated by RpoS were several involved in production of curli, an amyloid protein that functions as a structural component of biofilms (Salinas et al., 2020), proteins associated with biofilm formation, and the utilization of DNA as sole carbon source. The two-component system response regulator PhoP involved in stress response exhibited an enrichment of regulated genes in newer phylostrata, specifically the PS9 (Figure 4F). In this set of proteins, we find MgrB and SafA (formerly B1500) that both regulate PhoP activity (Lippa and Goulian, 2009; Eguchi et al., 2007). It could thus indicate that these evolutionary newer proteins have found a role in the fine-tuning of gene expression in the cell. Other proteins include ones with a role in acid stress response and regulation of intracellular magnesium ion concentration.

### 3.5 Younger genes tend to be related to developmental phenomena

**Figure 5:**
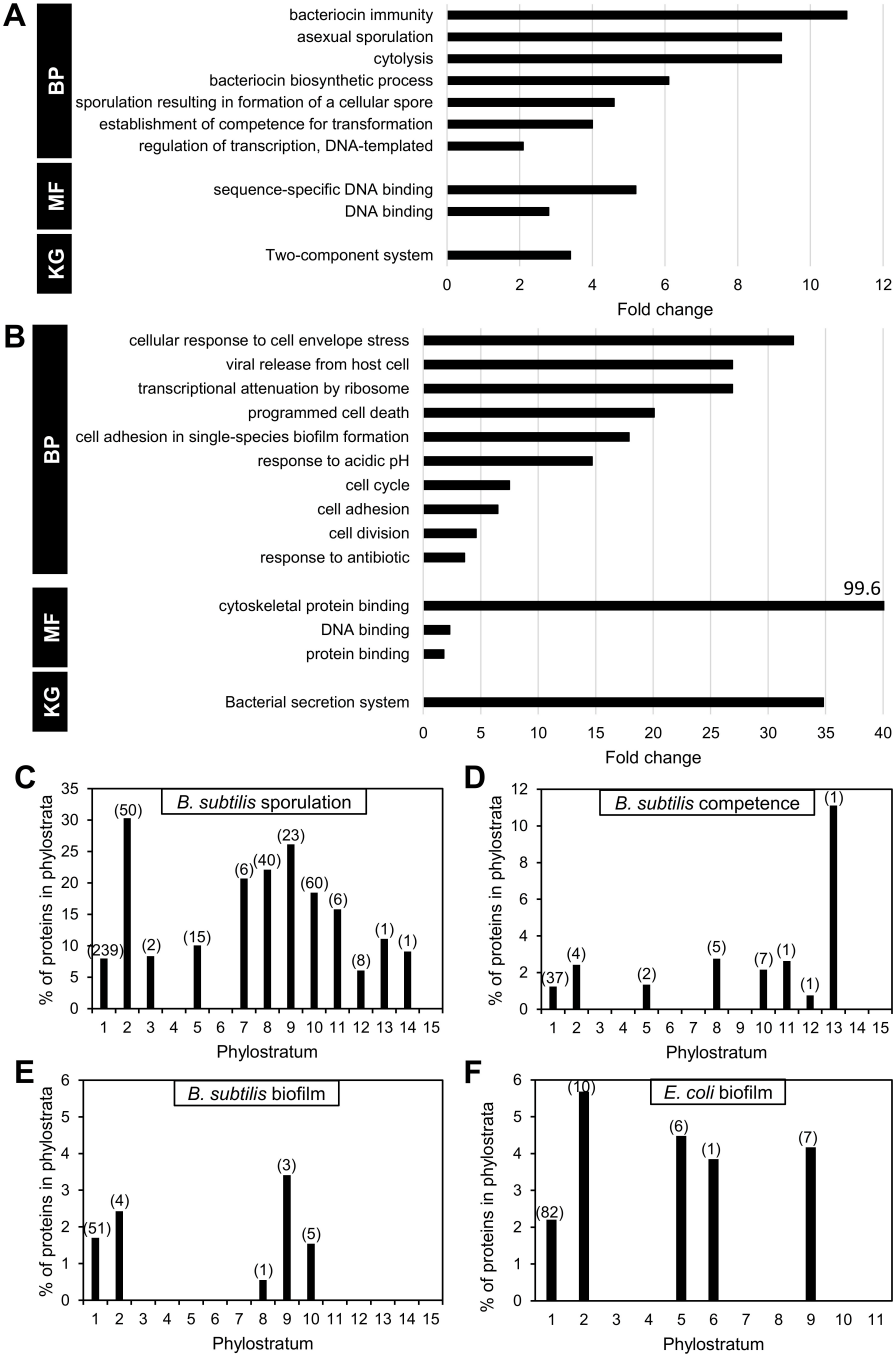
Over-representation analysis of proteins in later phylostrata. Functional over-representation based on the categories GO biological process (BP), Molecular function (MF), and KEGG (KG) terms for *B. subtilis* proteins in phylostrata 6–15 **(A)**, and *E. coli* proteins in phylostrata 6–11 **(B)**. GO terms showing very high enrichment but based on low gene counts (e.g., two or fewer genes) should be interpreted with caution as they may reflect chance associations rather than biologically significant trends. Phylostratigraphic age of proteins involved in developmental phenomena. Distribution in phylostrata of *B. subtilis* proteins involved in sporulation **(C)**, and genetic competence **(D)**. For biofilm formation, the distribution of proteins for *B. subtilis* and *E. coli* is shown **(E, F)**.

To provide an overview of the cellular processes that were developed later in evolution, we performed an over-representation analysis of GO and KEGG terms for the two bacteria. This was done both on the level of individual phylostrata as well as for the groups phylostrata 1–5 and 6–15/11. The rationale for the latter grouping of phylostrata is that the lineages of *B. subtilis* and *E. coli* diverged after PS5. Consequently, it could be argued that the genetic “innovation” differentiating between the two bacteria can be found primarily in phylostrata 6–15 (20% of genes) in *B. subtilis* and 6–11 (5.6% of genes) in *E. coli*. When performing the analysis on the set of older genes (PS1–5), over-represented categories included proteins in universal housekeeping processes such as “metabolic pathways,” “biosynthesis of secondary metabolites,” and “biosynthesis of Microbial metabolism in diverse environments” (Supplementary Data Sheet 5). In the newer phylostrata, in both bacteria, developmental programs were enriched (Figures 5A, B, Supplementary Data Sheet 5). In *B. subtilis*, genes involved in for examples sporulation, and genetic competence are over-represented. In the case of *E. coli*, different terms related to stress responses, cell division, as well as cell adhesion relevant for biofilm formation are over-represented. This prompted us to investigate development related genes more in depth.

Overall, majority of sporulation proteins belong to PS1 (53.9%). As previously reported (Shi et al., 2020), we found a significant involvement of younger genes in sporulation, especially in PS 7–11 (29.9%; Figure 5C). While sporulation presumably did not yet exist as a functional development program in PS1, a substantial number of older genes now participate in the process. These genes may have been functionally integrated into sporulation through regulatory or contextual changes, or possibly by undergoing sequence modification. A similar pattern was identified for other developmental phenomena, such as *B. subtilis* genetic competence (Figure 5D) and biofilm development (Figure 5E), as well as *E. coli* biofilm development (Figure 5F). In all these cases, the majority of developmental genes are found in PS1, but a substantial fraction of younger genes also contributes to these phenomena.

## 4 Discussion

What can be learned from looking at bacterial genome organization from the perspective of evolutionary age of genes? The bulk of bacterial genes are dedicated to core functions, including housekeeping, and these are naturally encoded by the oldest genes, situated in PS1–5 (Figures 1A, B, Supplementary Data Sheets 1, 2). A majority of the proteins in both species belonged to PS1 indicating that many of the core functions in present day bacteria also existed in the last universal common ancestor (LUCA). This contrasts with the situation in Eukarya, were the fraction of PS1 proteins is much lower, indicating that many more new genes appeared in this domain during the course of evolution. Developmental phenomena in bacteria, such as biofilm formation (Futo et al., 2021) and sporulation (Shi et al., 2020) are known to be more recent inventions, and as suggested by our study, typically involve newer genes (Figures 5C–F). This is in line with bacterial developmental processes being cited as models for ongoing social evolution in bacteria (Boyle et al., 2013). Since bacterial social interactions in biofilms are highly dynamic and evolvable, their intense evolution can be directly observed in adaptive evolution experiments (Martin et al., 2016). *B. subtilis* is often cited for its rich repertoire of developmental and community-based genetic programs, involving biofilm and pellicle formation, sporulation, cannibalism and genetic competence (Ricci-Tam et al., 2023). By contrast, *E. coli* has a less diverse lifestyle, with biofilm formation being the only developmental process it can do. This is very accurately reflected in the phylostratigraphy analysis, with only 5% of *E. coli* genes in PS6–11 (Figure 1B), and *B. subtilis* with 20% of all genes in PS6–15. Does this mean that *E. coli* evolved fewer *de novo* genes than *B. subtilis* during its evolution? Not necessarily. This question just brings us to horizontal gene transfer, recognized as a major generator of novelty in bacterial genomes (Arnold et al., 2022). The origins of novel bacterial genes likely involve a combination of evolutionary mechanisms. These include horizontal gene transfer from other bacteria or phages, gene duplication followed by divergence, frameshift-based innovation, and *de novo* emergence from previously non-coding sequences (Lang et al., 2017; Tautz and Domazet-Lošo, 2011; Neme and Tautz, 2014; Xia et al., 2025). While prophage regions represent identifiable hotspots for gene acquisition in both *E. coli* and *B. subtilis*, they account for only a fraction of the younger genes. Additional new genes may arise gradually within transcriptionally active regions, as proposed for proto-gene evolution in eukaryotes (Grandchamp et al., 2022), although the relative importance of these mechanisms in bacteria remains difficult to quantify. In many cases, phages represent a large fraction of the strain-specific DNA sequences (Brüssow et al., 2003). Our results suggest that the prophage regions in *E. coli* and *B. subtilis* genomes represent hot spots for more recent genes (Figure 3). Gene transfer is particularly intense in bacterial communities (Brito, 2021). This means that any *de novo* genes that evolve in bacteria became more or less immediately available to all community members by means of horizontal gene transfer. Then, the genetic makeup of an individual bacterial species gets defined by the preferred niche and environmental challenges. The soil dwelling *B. subtilis*, having to cope with vary variable and adverse environmental challenges, picked up more of the “novelty” tools for its adaptation toolbox. *E. coli*, adapting to a less challenging and more constant environment of a symbiont, does not require such a diverse developmental toolkit, hence the reduced proportion of novel, development-related genes. However, *E. coli* is fully capable of taking up additional functions when switching from commensal to pathogen lifestyle (Dobrindt et al., 2010), a phenomenon that has been described as the “unexhausted potential” of *E. coli* (Blount, 2015). Overall, it would appear that *de novo* genes are relatively accessible to bacteria, that are far less siloed than e.g., plants or metazoans, with strictly vertical evolution patterns.

Younger genes identified in bacteria follow the same pattern as those in more complex life forms (Neme and Tautz, 2014), they tend to be short, non-essential, and expressed to a low level or only under certain specific conditions. This description, in conjunction with the known association of essential and highly expressed genes to the bacterial origin of replication (Ying et al., 2014; Kosmidis et al., 2020; Lato and Golding, 2020), would suggest that the more recent genes should be expected to cluster closer to the replication terminus. Surprisingly, our analysis revealed a weak (with prophages included) or no correlation (with prophages excluded) between location of more recent genes and the replication origin (Figure 2). One possible explanation for this finding is linked to constraints on placement of new genes in the genome. As noted by Grandchamp et al. (2022), *de novo* genes in humans are more likely to arise in transcribed regions, coupled to existing elements of transcriptional regulation. This makes sense from the probabilistic perspective, since there are fewer constraints for creating an open reading frame from a non-coding sequence, than for creating a promoter region and other regulatory sequences required for ensuring gene expression in each organism. While bacteria do not have complex transcription regulation based on chromatin de-condensation to enable transcription, they do possess transcriptional regulatory units known as operons. We report an analogous observation that in bacteria new genes get predominantly inserted in preexisting operons (Figures 4A, B) and thus get placed under control of available transcription regulators (Figures 4C–F). As a housekeeping process, transcription control is generally ensured by old genes, and we found very few examples on new transcription regulators, such as ComK, Rok, GerR and SwrAA/1 from *B. subtilis*. Evolving of new developmental phenomena seldom involves establishing completely novel operons. Only 11 such operons were found in *B. subtilis* and 13 in *E. coli* (Tables 1, 2). Rather, new developmental programs in bacteria seem to arise as a combination of new genes, and extensive repurposing of older genes and operons (Figures 5C–F). This is supported by our observation that many of the genes involved in processes such as sporulation, competence, and biofilm formation originate from the oldest PS, particularly PS1, and have likely been incorporated into newer regulatory frameworks. This aligns with the view that bacterial developmental systems can evolve as modular additions, but also indicates that these modules are, at least in part, constructed from pre-existing components whose original functions were retained or adapted to new roles. In this light, bacterial development reflects both evolutionary innovation and the reorganization of existing genetic material, rather than purely the acquisition of entirely novel gene modules.

Our analysis of the two model bacteria suggests that there are important lessons to be learned from looking at bacterial genome evolution and genome organization from the perspective of evolutionary age of new genes. More recent bacterial genes tend to be short, non-essential, and their level of expression is generally low. Despite these features, newer genes are surprisingly not preferentially located far from the origin of replication. Their genomic location is rather uniform, and they are only in some instances enriched in areas containing mobile genetic elements, such as prophages. While most bacterial transcription regulators belong to the oldest phylostrata, they were found to regulate expression of both older and more recent genes alike. This suggests that many newer genes get inserted in the existing operons under the control of conserved regulatory elements. In particular, *E. coli* contains significantly fewer novel genes than *B. subtilis*, and this is mirrored by its more limited repertoire of developmental processes. By contrast, *B. subtilis* exhibits a more eventful evolutionary past in terms of acquisition of new genes, and particularly in this species, which possesses highly elaborate developmental regulation (e.g., during sporulation), it is evident that new regulatory systems have also evolved. The observation that many genetic operons contain genes from different phylostrata indicates a layered evolutionary history, in which new genes are incorporated into both pre-existing and newly formed frameworks for gene expression.

Genomic features described above suggest that phylostratigraphy approaches could assist in genome mining efforts (Mijakovic, 2020), not by predicting individual gene function directly, but by identifying phylostrata enriched in specific biological processes. For example, in the case of sporulation in *B. subtilis*, genes involved in this process were overrepresented in certain phylostrata, and a higher fraction of uncharacterized proteins from these phylostrata were experimentally shown to be involved in sporulation (Shi et al., 2020), demonstrating how phylostratigraphy can highlight candidate genes for targeted validation, particularly in biological processes involving functionally related gene sets that emerged during the same evolutionary interval.

## Data Availability

The original contributions presented in the study are included in the article/Supplementary material, further inquiries can be directed to the corresponding author.
