# Supplementary material for: Gene age and genome organization in Escherichia coli and Bacillus subtilis
Source: Front Microbiol. 2025 Jun 18;16:1512923. doi: 10.3389/fmicb.2025.1512923 (PMC12218254; doi:10.3389/fmicb.2025.1512923)
Supplement: Supplementary file 1 [file Table_1.pdf]

Supplementary table S1. Heterogenic operons without genes annotated as having unknown product or function. Gene name, PS and gene product (Subtiwiki) is listed and when subtiwiki description indicated a mechanistic role it is written in the comments field. For perons labelled red it is unclear how the operon could have had its function prior to introduction of the newer gene. For operons labelled green, the newer gene has a role in regulating the one or more of the older genes.

operon	Comments	genes								
atpI-atpC		atpI	atpB	atpE	atpF	atpH	atpA	atpG	atpD	atpC
		9	1	1	1	1	1	1	1	1
		ATP synthase (subunit i))	ATP synthase (subunit a))	ATP synthase (subunit c)	ATP synthase (subunit b))	ATP synthase (subunit delta)	ATP synthase (subunit alpha)	ATP synthase (subunit gamma))	ATP synthase (subunit beta))	ATP synthase (subunit epsilon))
comGA -spoIIIL		comGA	comGB	comGC	comGD	comGE	comGF	comGG	spoIIIL	
		1	1	2	8	10	10	10	9	
		traffic ATPase	DNA transport machinery	major pseudopilin	minor pseudopilin	minor pseudopilin	DNA transport machinery	minor pseudopilin	component of the [[protein|SpoIIIA]]-[[protein|SpoIIQ]] trans-envelope complex	.
spoIIIAA-spoIIIAH		spoIIIAA	spoIIIAB	spoIIIAC	spoIIIAD	spoIIIAE	spoIIIAF	spoIIIAG	spoIIIAH	
		1	8	2	2	1	8	8	1	
		AAA protease	component of a type III secretion system	component of a type III secretion system	component of a type III secretion system	component of a type III secretion system	component of a type III secretion system	component of a type III secretion system	part of the transmembrane channel linking the mother cell and the forespore	.
maf-minD	mreD (PS9) is a cell shape-determing protein, essential under normal conditions	maf	radC	mreB	mreC	mreD	minC	minD		
		1	1	1	1	9	1	1		
		nucleotide pyrophosphatase	probable DNA repair protein	[SW|cell shape]-determining protein	cell shape-determining protein	cell shape-determining protein	cell-division inhibitor	[SW|cell division] inhibitor		
walR-htrC	yycH (PS9) and yycI (PS8) are regulators of sensor kinase walK	walR	walK	yycH	yycI	walJ	htrC			
		1	1	9	8	1	1			
		two-component response regulator ([SW|OmpR family])	two-component sensor kinase (OmpR family)	negative effector of [[protein|WalK]]	negative effector of [[protein|WalK]]	5'-3' double-stranded DNA exonuclease	putative quality control membrane protease			.
yknU-yknZ	yknW (PS9) modulates assembly of the YknX-YknY-YknZ ABC transporter for the export of the SdpC toxin	yknU	yknV	yknW	yknX	yknY	yknZ			
		1	1	9	1	1	1			
		[SW|ABC transporter] (ATP-binding protein)	[SW|ABC transporter] (ATP-binding protein)	modulator of ABC transporter assembly	ABC-type antimicrobial peptide exporter (permease)	[SW|ABC transporter](ATP-binding protein, exporter)	[SW|ABC transporter] (permease)			.
bacA-bacF		bacA	bacB	bacC	bacD	bacE	bacF			
		5	1	1	1	1	1			
		anticapsin biosynthesis protein, prephenate decarboxylase	oxidase	dihydroanticapsin 7-dehydrogenase	alanine-anticapsin ligase	efflux protein for bacilysin excretion	aminotransferase			

hemA-hemL	Heme biosynthesis by PS1 proteins in operon with PS5 protein with likely regulatory role	hemA	hemX	hemC	hemD	hemB	hemL			
		1	5	1	1	1	1			
		glutamyl-tRNA reductase	regulatory protein, protease?	hydroxymethylbilane synthase	uroporphyrinogen-III synthase	porphobilinogen synthase	glutamate-1-semialdehyde aminotransferase			.
hutP-hutM	hutP (PS4) regulates the expression of the hut histidine utilization operon	hutP	hutH	hutU	hutI	hutG	hutM			
		4	1	1	1	1	1			
		transcriptional antiterminator	histidase	urocanase	imidazolone-5-propionate hydrolase	formiminoglutamate hydrolase	histidine permease			
ctsR-disA	McsA (PS2) regulates McsB arginine kinase and CtsR (PS2) that in turn regulate expression of the operon	ctsR	mcsA	mcsB	clpC	radA	disA			
		2	2	1	1	1	1			
		transcription repressor	activator of [[protein|McsB]] kinase activity	protein arginine kinase	AAA unfoldase, ATPase subunit of the [[protein|ClpC]]-[[protein|ClpP]] protease	branch migration transferase, 6-O-methylguanine-DNA methyltransferase	DNA integrity scanning protein, has diadenylate cyclase activity			.
srfAA-srfAD		srfAA	srfAB	comS	srfAC	srfAD				
		1	1	13	1	1				
		surfactin synthetase / competence	surfactin synthetase / competence	antiadaptor protein (anti-MecA)	surfactin synthetase / competence	surfactin synthetase / competence				.
sunA-bdbB	SunA (PS12) encode sublancin precursor peptide and is in operon with proteins involved in its synthesis and export	sunA	sunT	bdbA	yolJ	bdbB				
		12	1	1	1	1				
		sublancin 168 lantibiotic antimicrobial precursor peptide	[[protein|SunA|sublancin]] 168 lantibiotic [SW|ABC transporter]	thiol-disulfide oxidoreductase	sublancin S-glycosyltransferase	thiol-disulfide oxidoreductase				
yydF-yydJ		yydF	yydG	yydH	yydI	yydJ				
		9	1	2	1	8				
		secreted peptide	radical SAM epimerase	membrane protease	[SW|ABC transporter], ATP-binding protein	[SW|ABC transporter], permease				.
pucA-pucE		pucA	pucB	pucC	pucD	pucE				
		1	1	5	1	1				
		xanthine dehydrogenase	xanthine dehydrogenase	xanthine dehydrogenase	xanthine dehydrogenase	xanthine dehydrogenase				
fapR-acpA	fapR (PS4) is a transcriptional repressor of the fatty acid biosynthesis operon	fapR	plsX	fabD	fabG	acpA				
		4	1	1	1	1				
		transcriptional repressor	acyl-acyl carrier protein (ACP):phosphate acyltransferase	malonyl CoA-acyl carrier protein transacylase	beta-ketoacyl-acyl carrier protein reductase	acyl carrier protein				
ribD-ribT	RibT (PS3) regulates RibH by acetylation	ribD	ribE	ribA	ribH	ribT				
		1	1	1	1	3				

		5-amino-6-(5-phosphoribosylamino)uracil reductase	riboflavin synthase (alpha subunit)	GTP cyclohydrolase II/ 3,4-dihydroxy-2-butanone 4-phosphate synthase	riboflavin synthase (beta subunit)	dioxin reductive etherase				
yaaA-gyrA		yaaA	recF	remB	gyrB	gyrA				
		1	1	2	1	1				
		ribosome assembly factor	promoter of [[protein|RecA]] DNA repair center assembly	regulator of the extracellular matrix	DNA gyrase (subunit B)	DNA gyrase (subunit A)				
spoIVFA-rpmA	spoIVFA (PS2) inhbits spoIVFB and prp (PS2) is a protease required for maturation of L27	spoIVFA	spoIVFB	rplU	prp	rpmA				
		2	1	1	2	1				
		inhibitor of SpoIVFB metalloprotease	intramembrane metalloprotease	ribosomal protein L21 (BL20)	[[protein|RpmA|ribosomal protein L27]] cysteine protease	ribosomal protein L27 (BL24)				
yfkQ-yfkT		yfkQ	yfkR	yfkS	yfkT					
		1	2	11	2					
		part of the YfkQ-YfkR-YfkT germinant receptor	part of the YfkQ-YfkR-YfkT germinant receptor	D protein for the YfkQ-YfkR-YfkT germinant receptor	part of the YfkQ-YfkR-YfkT germinant receptor					.
flgM-flgL	yvyG (PS10) is required for export of the hook-filament junction proteins, FlgK and FlgL	flgM	yvyG	flgK	flgL					
		1	10	1	1					
		anti-[[protein|SigD]]	flagellar filament assembly protein	flagellar hook-filament junction proteins	flagellar hook-filament junction proteins					.
yknW-yknZ	yknW (PS9) modulates assembly of ABC transporter yknXYZ	yknW	yknX	yknY	yknZ					
		9	1	1	1					
		modulator of ABC transporter assembly	ABC-type antimicrobial peptide exporter (permease)	[SW|ABC transporter](ATP-binding protein, exporter)	[SW|ABC transporter] (permease)					.
spo0B-pheA		spo0B	obg	thrR	pheA					
		8	1	6	1					
		[SW|sporulation]initiation phosphotransferase	GTP-binding protein	transcription repressor of threonine biosynthetic gene	prephenate dehydratase					
ydiG-tatCY		ydiG	rex	tatAY	tatCY					
		1	1	8	1					
		molybdenum cofactor biosynthesis protein	transcriptional repressor	component of the twin-arginine translocation pathway	component of the twin-arginine translocation pathway					.
qoxA-qoxD	qoxD (PS5) a subunit in the qoxABCD	qoxA	qoxB	qoxC	qoxD					
		1	1	1	5					

<table>
<tr><td></td><td>cytochrome aa3 quinol oxidase</td><td>cytochrome aa3 quinol oxidase (subunit II)</td><td>cytochrome aa3 quinol oxidase (subunit I)</td><td>cytochrome aa3 quinol oxidase (subunit III)</td><td>cytochrome aa3 quinol oxidase (subunit IV)</td><td></td><td></td><td></td><td></td><td></td><td>.</td></tr>
<tr><td>rpmH-jag</td><td></td><td>rpmH</td><td>rnpA</td><td>spoIIIJ</td><td>jag</td><td></td><td></td><td></td><td></td><td></td><td></td></tr>
<tr><td></td><td></td><td>2</td><td>2</td><td>1</td><td>1</td><td></td><td></td><td></td><td></td><td></td><td></td></tr>
<tr><td></td><td></td><td>ribosomal protein L34</td><td>protein component of RNAse P (substrate specificity)</td><td>membrane protein translocase</td><td>[[protein|SpoIIIJ]]-associated protein</td><td></td><td></td><td></td><td></td><td></td><td>.</td></tr>
<tr><td>polA->ytaG</td><td></td><td>polA</td><td>mutM</td><td>ytaF</td><td>ytaG</td><td></td><td></td><td></td><td></td><td></td><td></td></tr>
<tr><td></td><td></td><td>1</td><td>1</td><td>2</td><td>1</td><td></td><td></td><td></td><td></td><td></td><td></td></tr>
<tr><td></td><td></td><td>DNA polymerase I</td><td>formamidopyrimidine-DNA glycosidase</td><td>[SW|sporulation] protein</td><td>dephospho-CoA kinase</td><td></td><td></td><td></td><td></td><td></td><td></td></tr>
<tr><td>usd-mbl</td><td>Usd (PS13) required for translation of SpoIIID</td><td>usd</td><td>spoIIID</td><td>mbl</td><td></td><td></td><td></td><td></td><td></td><td></td><td></td></tr>
<tr><td></td><td></td><td>13</td><td>2</td><td>1</td><td></td><td></td><td></td><td></td><td></td><td></td><td></td></tr>
<tr><td></td><td></td><td>putative spoIIID leader peptide</td><td>transcriptional regulator</td><td>[[protein|MreB]]-like protein</td><td></td><td></td><td></td><td></td><td></td><td></td><td></td></tr>
<tr><td>bmrB-bmrD</td><td>bmrB (PS12) is a regulatory leader peptide for the control of bmrC-bmrD expression</td><td>bmrB</td><td>bmrC</td><td>bmrD</td><td></td><td></td><td></td><td></td><td></td><td></td><td></td></tr>
<tr><td></td><td></td><td>12</td><td>1</td><td>1</td><td></td><td></td><td></td><td></td><td></td><td></td><td></td></tr>
<tr><td></td><td></td><td>leader peptide</td><td>multidrug [SW|ABC transporter](ATP-binding protein)</td><td>multidrug [SW|ABC transporter](ATP-binding protein)</td><td></td><td></td><td></td><td></td><td></td><td></td><td>.</td></tr>
<tr><td>khtS-khtU</td><td>K+/H+ antiporter composed of two PS1 proteins and regulated by PS10 protein</td><td>khtS</td><td>khtT</td><td>khtU</td><td></td><td></td><td></td><td></td><td></td><td></td><td></td></tr>
<tr><td></td><td></td><td>10</td><td>1</td><td>1</td><td></td><td></td><td></td><td></td><td></td><td></td><td></td></tr>
<tr><td></td><td></td><td>modulator of YhaU activity</td><td>K /H antiporter for K efflux</td><td>K⁺/H⁺ antiporter for K⁺ efflux</td><td></td><td></td><td></td><td></td><td></td><td>.</td><td></td></tr>
<tr><td>tapA-tasA</td><td></td><td>tapA</td><td>sipW</td><td>tasA</td><td></td><td></td><td></td><td></td><td></td><td></td><td></td></tr>
<tr><td></td><td></td><td>10</td><td>1</td><td>9</td><td></td><td></td><td></td><td></td><td></td><td></td><td></td></tr>
<tr><td></td><td></td><td>[[protein|TasA]] anchoring/assembly protein</td><td>signal peptidase I</td><td>major component of biofilm matrix</td><td></td><td></td><td></td><td></td><td></td><td></td><td>.</td></tr>
<tr><td>sigM-yhdK</td><td>SigM (PS1) is found in operon with two regulators of higher PS</td><td>sigM</td><td>yhdL</td><td>yhdK</td><td></td><td></td><td></td><td></td><td></td><td></td><td></td></tr>
<tr><td></td><td></td><td>1</td><td>8</td><td>10</td><td></td><td></td><td></td><td></td><td></td><td></td><td></td></tr>
<tr><td></td><td></td><td>[SW|RNA polymerase] ECF-type [SW|sigma factor] SigM</td><td>anti-[[protein|SigM]] protein</td><td>anti-[[protein|SigM]] protein</td><td></td><td></td><td></td><td></td><td></td><td></td><td></td></tr>
<tr><td>scpA-ypuI</td><td></td><td>scpA</td><td>scpB</td><td>ypuI</td><td></td><td></td><td></td><td></td><td></td><td></td><td></td></tr>
<tr><td></td><td></td><td>1</td><td>1</td><td>10</td><td></td><td></td><td></td><td></td><td></td><td></td><td></td></tr>
<tr><td></td><td></td><td>DNA segregation and condensation protein</td><td>DNA segregation and condensation protein</td><td>rRNA pseudouridine 2633 synthase</td><td></td><td></td><td></td><td></td><td></td><td></td><td>.</td></tr>
<tr><td></td><td></td><td>yqeZ</td><td>floA</td><td>yqfB</td><td></td><td></td><td></td><td></td><td></td><td></td><td></td></tr>
</table>

yqeZ-yqfB		1	1	10						
		NfeD family protein NfeD1b, serine protease	flottilin-like protein	resistence protein (against sublancin)						.
cotSA-ytxO		cotSA	cotS	ytxO						
		1	8	10						
		spore coat protein	spore coat protein	outer spore coat protein						
mutY-sspE		mutY	fabL	sspE						
		1	1	9						
		A/G-specific adenine glycosylase	enoyl-acyl carrier protein reductase	small acid-soluble spore protein (major gamma-type SASP)						.
ctc-fin		ctc	spoVC	fin						
		1	1	8						
		ribosomal protein	peptidyl-tRNA hydrolase	feedback inhibitor of [[protein|SigF]] activity						.
lytA-lytC		lytA	lytB	lytC						
		8	1	1						
		secretion of major autolysin LytC	modifier protein of major autolysin LytC	N-acetylmuramoyl-L-alanine amidase						
alr-ndoA	ndoAI (MazE)(PS8) is an anti-toxin that inactives ndoA (MazF).	alr	ndoAI	ndoA						
		1	8	1						
		alanine racemase	antitoxin	mRNA interferase						
sigH-secE		sigH	rpmGB	secE						
		1	1	8						
		[SW|RNA polymerase] [SW|sigma factor] SigH	ribosomal protein L33b	preprotein translocase subunit						.
fin-spoVT		fin	mfd	spoVT						
		8	1	2						
		feedback inhibitor of [[protein|SigF]] activity	[SW|transcription]-repair coupling factor	transcriptional regulator						
cotH-ywrJ		cotH	cotB	ywrJ						
		1	8	8						
		protein kinase	spore coat protein (outer)	sporulation protein						
immR-int	immA (PS5) is a protease that degrades and thereby regulate	immR	immA	int						
		1	5	1						

	transcriptional repressor immR	transcriptional repressor ([SW|Xre family])	site-specific protease	integrase						
sdhC-sdhB		sdhC	sdhA	sdhB						
		5	1	1						
		succinate dehydrogenase (cytochrome b558 subunit)	succinate dehydrogenase (flavoprotein subunit)	succinate dehydrogenase (iron-sulfur protein)						.
aroF-aroH		aroF	aroB	aroH						
		1	1	2						
		chorismate synthase	3-dehydroquinate synthase	chorismate mutase (isozymes 1 and 2)						
cotV-cotX		cotV	cotW	cotX						
		10	10	9						
		spore crust protein (insoluble fraction)	spore crust protein (insoluble fraction)	spore crust protein (insoluble fraction)						.
gerAA-gerAC	GerAC (PS2) part of the nutrient-gated GerAA-GerAB-GerAC ion channel	gerAA	gerAB	gerAC						
		1	1	2						
		nutrient receptor	nutrient receptor	nutrient receptor						
gerBA-gerBC	GerBC (PS2) part of the putative nutrient-gated GerBA-GerBB-GerBC ion channel	gerBA	gerBB	gerBC						
		1	1	2						
		nutrient receptor	nutrient receptor	nutrient receptor						
spoIIGA-sigG	spoIIGA (PS2) required for processing of SigE	spoIIGA	sigE	sigG						
		2	1	1						
		Pro-[[protein|SigE]] protease	[SW|RNA polymerase][SW|sporulation]mother cell-specific (early) [SW|sigma factor]SigE	[SW|RNA polymerase] [SW|sporulation] forespore-specific (late) [SW|sigma factor] SigG					.	
dacB-spmB		dacB	spmA	spmB						
		1	2	2						
		penicillin-binding protein 5*, D-alanyl-D-alanine carboxypeptidase	spore maturation protein (spore core dehydratation)	spore maturation protein (spore core dehydratation)						.
yndD-yndF	YndF (PS2) is part of the YndD-YndE-YndF germinant receptor of unknown specificity	yndD	yndE	yndF						
		1	1	2						
		part of the YndD-YndE-YndF germinant receptor	part of the YndD-YndE-YndF germinant receptor	part of the YndD-YndE-YndF germinant receptor						.
