# Supplementary figures and images for: Gene age and genome organization in Escherichia coli and Bacillus subtilis
Source: Front Microbiol. 2025 Jun 18;16:1512923. doi: 10.3389/fmicb.2025.1512923 (PMC12218254; doi:10.3389/fmicb.2025.1512923)

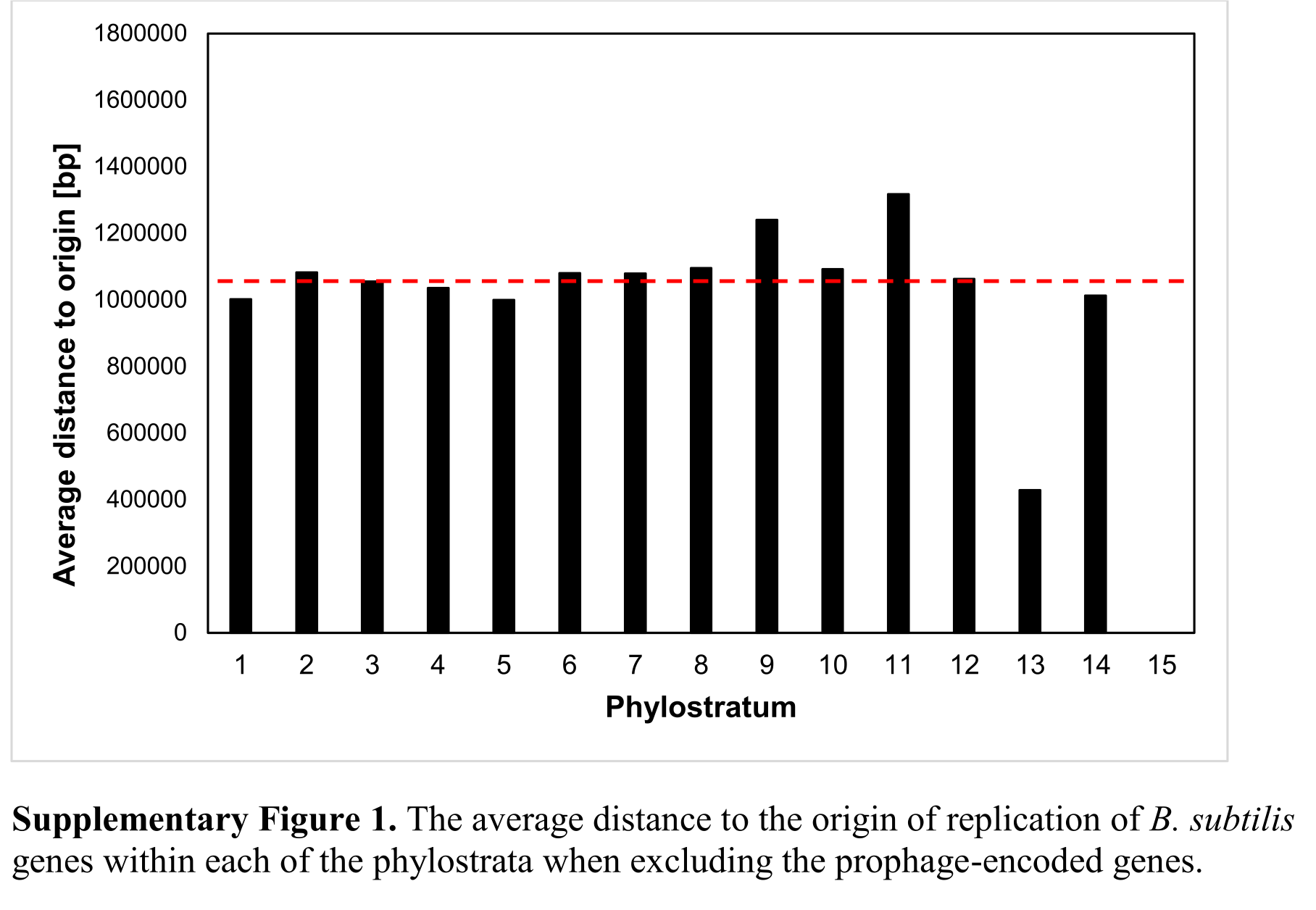

Supplement: Supplementary file 7 [file Image_1.tif]

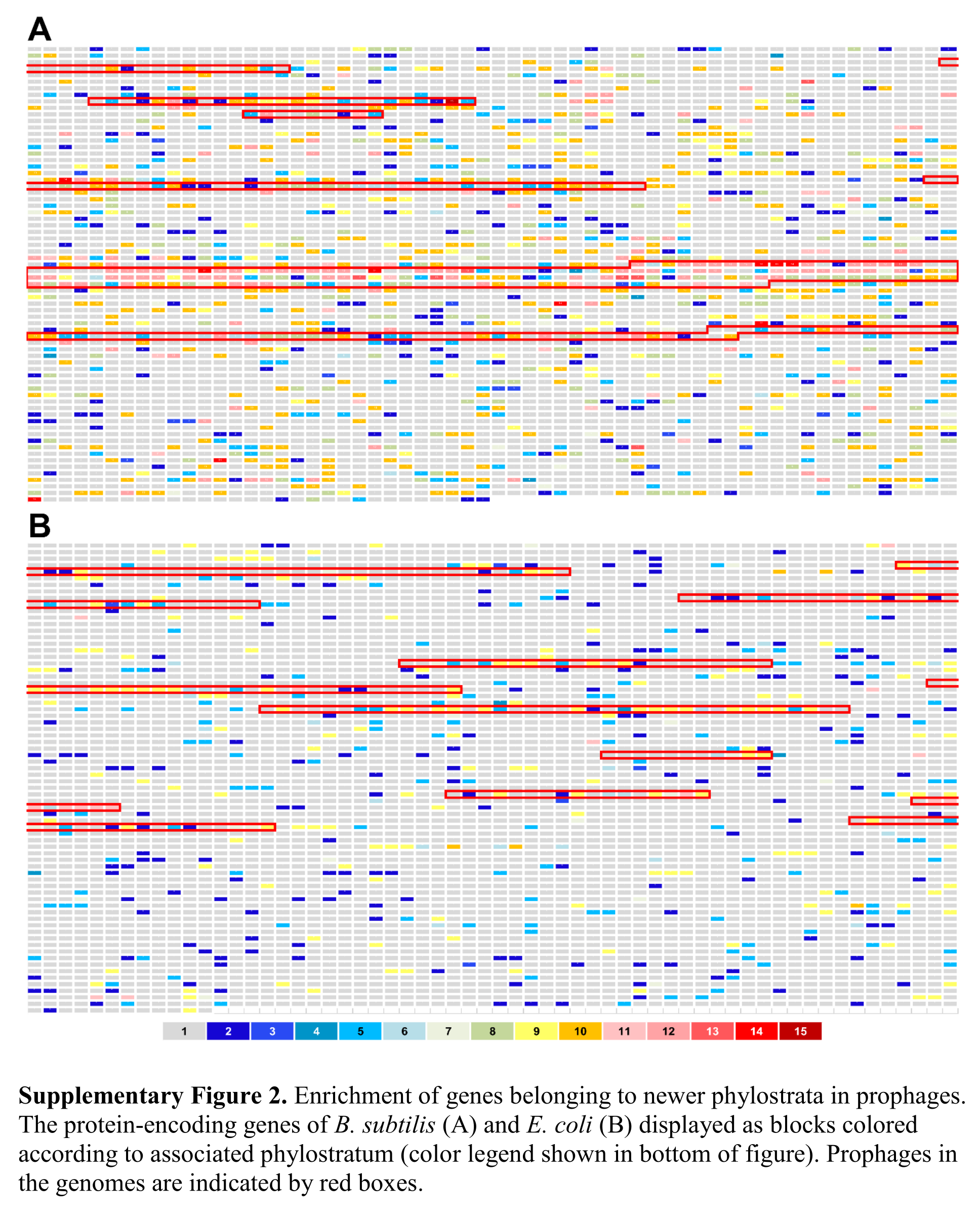

Supplement: Supplementary file 8 [file Image_2.tif]

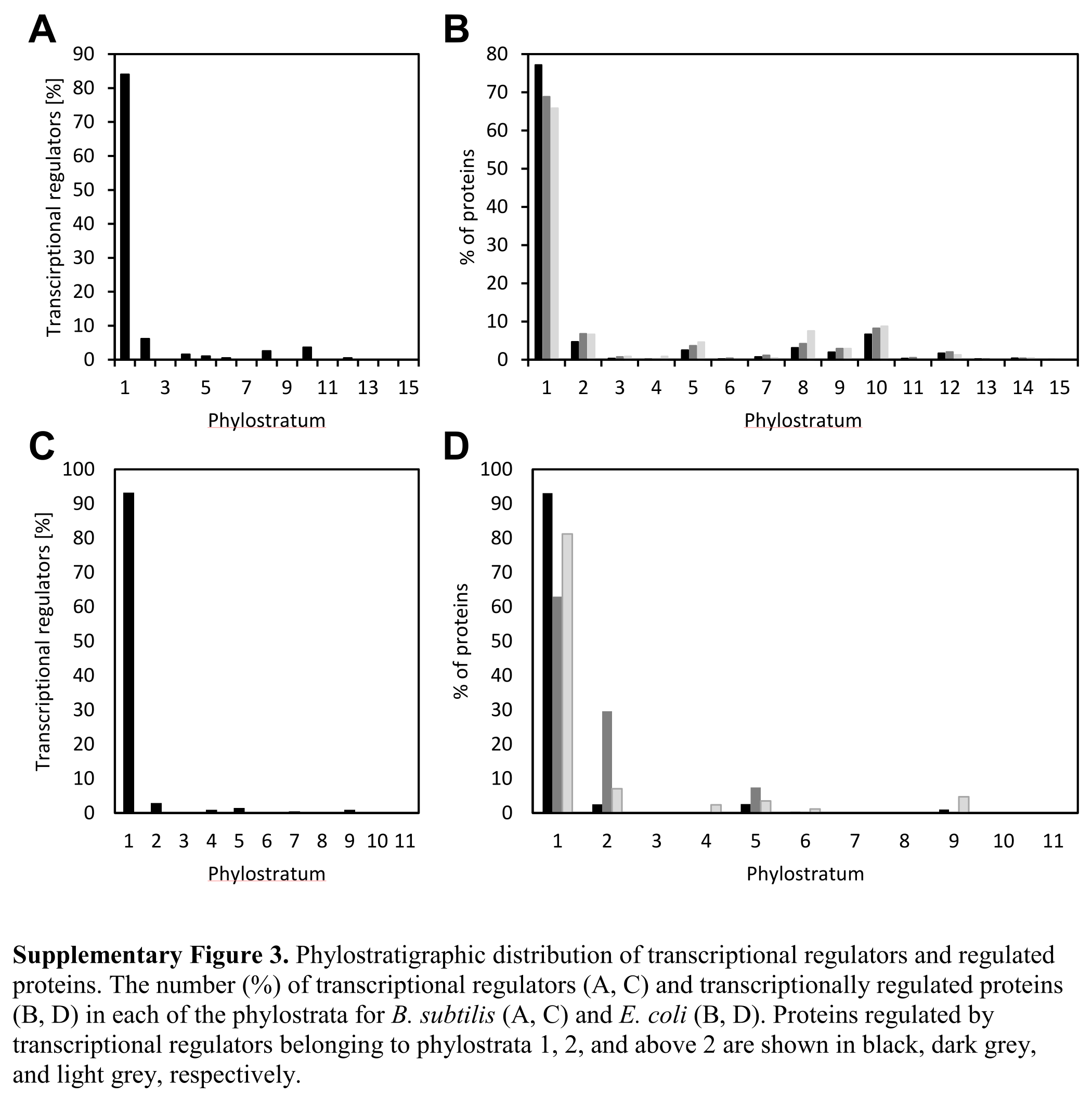

Supplement: Supplementary file 9 [file Image_3.tif]
